# Chiral method validation and stereoselective degradation of profoxydim isomers in paddy soil

**DOI:** 10.1007/s11356-024-35557-z

**Published:** 2024-11-20

**Authors:** Álvaro Cervantes-Díaz, Miguelina Mateo-Miranda, José Luis Alonso-Prados, Pilar Sandín-España

**Affiliations:** 1 Plant Protection Products Unit/Plant Protection Department, National Institute for Agricultural and Food Research and Technology INIA-CSIC, Ctra. La Coruña, Km. 7.5, 28040 Madrid, Spain; 2 Department of Agricultural Chemistry and Food Science, UAM, Madrid, Spain

**Keywords:** Profoxydim herbicide, HPLC–MS/MS, QuEChERS, Stereoisomers, Degradation, Soil

## Abstract

The herbicide profoxydim, which is chiral in nature, is commonly used for weed control in rice. However, traditional achiral analysis treats profoxydim isomers as a single compound, providing only partial and inaccuracy information assuming identical behavior for the four isomers. To address this issue, a sensitive and straightforward chiral analytical method was developed using QuEChERS and chiral HPLC–MS/MS to determine the residues of all four stereoisomers of profoxydim in soil. The method was validated and found to have good accuracy and precision, with recoveries ranging from 88.27 to 104.93% for dry soil and from 98.25 to 106.53% for soaked soil, with RSD values lower than 11.14%. The limit of detection for the profoxydim isomers was 7.6 µg/kg, and the limit of quantification was 25.0 µg/kg. Furthermore, the proposed method was employed for the first time to investigate the individual degradation of novel isolated stereoisomers of profoxydim in soil and the kinetics and evolution of the generated degradation products (DPs). The degradation of the four stereoisomers followed first-order kinetics and was found to be stereoselective, with isomers 1 and 4 preferentially degrading with half-lives of 14.7 and 15.6 h respectively. The profoxydim enantiomers remained configurationally stable without any interconversion throughout the experiment. The findings of this study provide a robust scientific basis for improving profoxydim risk assessment and highlight the need to characterize chiral agrochemical isomers to better understand their environmental distribution and fate.

**Supplementary Information:**

The online version contains supplementary material available at 10.1007/s11356-024-35557-z.

## Introduction

Pesticide chirality is an emerging area of research in agricultural and environmental chemistry, mainly considering that more than 40% of the pesticides used are chiral (Jeschke 2018; Lucci et al. 2022). The fate and behavior of these pesticides and their metabolites are of great interest and concern. It is known that enantiomers of chiral pesticides have identical physical and chemical properties but they can differ significantly in their environmental fate when interacting with other chiral molecules, such as enzymes and biological receptors of microorganisms present, for instance, in water or soils (Buerge et al. 2003; Garrison 2006; Qu et al. 2014). Stereoselective degradation in soils has been observed for various chiral pesticides (Castineira-Landeira et al. 2023; Hazra et al. 2023). Ramezani et al. (2010) observed that the R enantiomer of several herbicides of the imidazole family, which had higher herbicidal activity (up to eightfold), degraded more rapidly than the less active S enantiomer. Furthermore, the biological activity of the individual isomers can also be different, resulting in toxicity and efficacy. Thus, pesticides of the triazole family showed a completely different biological activity between the two stereoisomers; for example, R isomer of diniconazole is a fungicide whereas the S isomer is a plant growth regulator (Magrans et al. 2002). In the case of the triadimenol fungicide, the 1S2R stereoisomer was shown to be up to 1000 times more active than the other three stereoisomers (Burden et al. 1987). Regarding the bioactivity and toxicity, the R isomer of the herbicide lactofen is the active one and is easily degraded in the environment, while the S isomer is more persistent in soil and 50 times more toxic the aquatic flea to *D. magna* and the aquatic algae (*Microcystis aeruginosa*) (Xie et al. 2018).

However, to date, most studies on chiral pesticides do not consider individual stereoisomers, even though they may exhibit different biological behaviors. In fact, only 7% of chiral pesticides are currently commercialized as pure stereoisomers or as fortified formulations of the active stereoisomer (Garrison et al. 2012). As a result, current knowledge of these chiral pollutants is often inaccurate because it incorrectly assumes that stereoisomers have identical environmental behavior. Conventional achiral analyses cannot measure enantiomeric contents, but only one chromatographic peak which is the sum of all the stereoisomers. In the event that a more toxic stereoisomer is preferentially degraded, the results of exposure assessments based on achiral concentration measurements will be an overestimation of toxicity. Conversely, if less hazardous stereoisomers are preferentially eliminated, toxicity based on achiral measures will be underestimated. Therefore, due to the widespread use of chiral pesticides in modern agriculture, it is necessary to study chiral pesticide residues and their environmental behavior at the stereoisomeric level.

All this knowledge could potentiate the use of enantiomerically pure chiral pesticides, reducing the risks associated with pesticide exposure (Lucci et al. 2022). This could also help meet the European Commission’s target of reducing pesticide use and associated risks by 50% by 2030 (European Commission 2020).

Soil is a complex medium and one of the most challenging matrices in the environment for pesticide extraction and quantification. Agricultural soils have different and widely varied chemical and physical compositions. Organic and mineral colloids have a profound influence on pesticide adsorption and subsequent extraction efficiency (Spark and Swift 2002). Pesticide extraction from soils has been thoroughly studied, and conventional extraction methods (e.g., Soxhlet extraction) have been replaced by greener and faster techniques such as ultrasound-assisted extraction (UAE) (Geronimo et al. 2015; Castineira-Landeira et al. 2023), pressurized liquid extraction (PLE) (Vidal et al. 2010; Mustafa and Turner 2011), vortex extraction (VE) (Guerra et al. 2018), and microwave-assisted extraction (MAE) (Fuentes et al. 2007; Zhang et al. 2012). Currently, the search for sustainable and environmentally friendly techniques is highly important. The introduction of the QuEChERS method (quick, easy, cheap, effective, rugged, safe) over the last few decades has significantly increased the versatility of soil sample analysis (Caldas et al. 2011; Brinco et al. 2023; Liu et al. 2024).

**Fig. 1:**
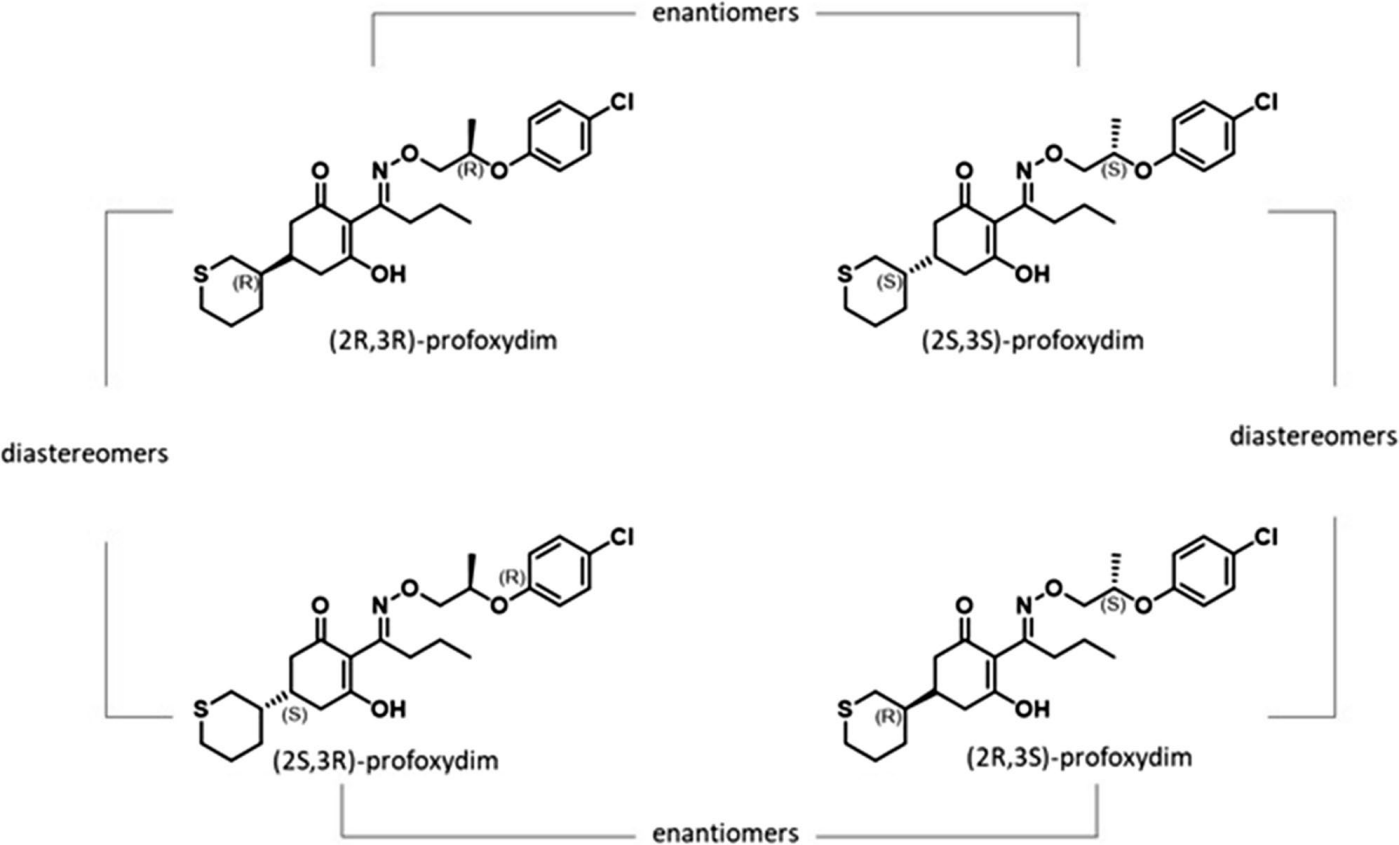
Chemical structures of stereoisomers of profoxydim

Among chiral pesticides, profoxydim is a last-generation cyclohexanedione herbicide that is used for the postemergence control of grassy weeds in rice. The compound has *E/Z* isomerism and two chiral carbons. The herbicide is commercially available as the E isomer and a mixture of the four stereoisomers (25% each) (Fig. 1). The presence of multiple stereoisomers presents a challenge to chemical analysis, degradation studies, and risk assessment.

It is well documented that profoxydim has a low persistence, given that it is readily degraded under environmental conditions and breaks down easily. Sanchez et al. (2006), Tsochatzis et al. (2013), and Cervantes-Diaz et al. (2024) have reported the low stability of this herbicide in paddy soils, with half-lives between 3 and 13 days. However, these studies considered profoxydim as a mixture of isomers, which means that the results are not accurate enough as they represent the average half-lives of all isomers. Moreover, the analytical techniques employed in these studies did not differentiate between the stereoisomers of profoxydim, instead treating them as a single entity. To our knowledge, the environmental fate of individual profoxydim stereoisomers in the soil is still unknown. Thus, it is essential to obtain stereospecific information on the chirality of profoxydim to make a more accurate benefit-risk evaluation. To achieve this goal, it is necessary to develop a chiral method for the separation and isolation of profoxydim stereoisomer residues in soil and to obtain information related to the degradation of each stereoisomer. Although a previous chiral method was validated in rice (Cervantes-Díaz et al. 2024), the development of a method in such a different and complex compartment as soil and the isolation of each isomer have been achieved for the first time in this work.

The aim of the present study was to (a) develop and validate a highly sensitive analytical method for the determination of profoxydim isomers in soil by chiral HPLC–MS/MS, (b) investigate the degradation of each isolated stereoisomers in paddy soil, and (c) identify DPs. The final objective of this study was to illustrate the environmental behavior of profoxydim at the isomer and byproduct levels. The results provide new insights into the stereoisomeric behavior of profoxydim, which will be of significant value in enhancing our comprehension of the fate of chiral pesticides in the environment.

## Materials and methods

### Chemicals and materials

The analytical standard of profoxydim ((1EZ)-N-[(2RS)-2-(4-chlorophenoxy)propoxy]butanimidoyl)-3-hydroxy-5-[(3RS)-thian-3-yl]cyclohex-2-en-1-one) (98.8% purity), marketed as the *E* isomer, was acquired from HPC Standards GmbH (Cunnersdorf, Deutschland). Its properties were 466.1 g/mol of molecular weight, water solubility of 531 mg/L, log Pow of 3.7 at 25 °C, 1.7 × 10^−1^ mPa vapor pressure at 20 °C, and a pKa of 5.9 at 20 °C (MacBean 2012). Acetonitrile and methanol (HPLC–MS grade) were purchased from Macron Fine Chemicals (Gliwice, Poland) and formic acid from Scharlab S.L. (Barcelona, Spain).

A solution of 100 µg/mL of profoxydim dissolved in acetonitrile was employed to prepare more diluted stock solutions for method development and validation studies. The solutions were maintained at 4 °C in the dark.

To isolate each profoxydim isomer, solid phase extraction (SPE) was used to extract and concentrate the compounds. Two types of SPE cartridges were tested: Isolute ENV + and octadecyl C18 cartridges. The samples were passed through the cartridges under a vacuum provided by a 12-port Visiprep SPE Vacuum manifold from Supelco (Supelco, Bellefonte, PA, USA).

Three QuEChERS methods were evaluated including the citrate-buffered method, acetate-buffered method, and non-buffered method (see Supplementary Information section for detailed information regarding the salts and quantities used).

Regarding the d-SPE clean-up step, various dispersive adsorbents based on graphitized carbon black (GCB), primary secondary amine (PSA), and octadecylsilane (C18) were evaluated (see Supplementary Information section for detailed information on adsorbents).

A vortex stirrer from BioCote (Coventry, UK) and an ultrasound bath from J.P. Selecta (Barcelona, Spain) were used for VE and UAE. Agitation of the QuEChERS tubes was performed by using a multivortex BenchMixer XL (Benchmark Scientific, USA) to avoid possible deviations due to manual agitation. Eppendorf 5810 centrifuge (Eppendorf Iberica, Hamburg, Germany) was used to centrifuge the samples.

Paddy soil was collected from a Seville rice field (37° 12′ 27.4″ N 5°49′ 13.1″ W) in southern of Spain. A total of 20 samples were obtained from the rice field and subsequently combined to form a single, representative sample, which was then used for the present studies. The soil was stored at 4 °C, air-dried overnight, and passed through a 2-mm sieve before use. Then, the soil was checked for the presence of profoxydim. As expected, no detectable residues of profoxydim were detected.

### Sample preparation and chromatographic analysis

A stock solution of profoxydim was prepared and added to fortified soil samples (4 g) that had been weighed into centrifuge tubes (50 mL). Samples and standards were combined meticulously and allowed to rest for 1 h before extraction. This allowed the herbicide to permeate the matrix effectively. Acetonitrile solution (10 mL) was then added, and the tube was shaken for 1 min with a vortex mixer. Subsequently, non-buffered QuEChERS was added in order to induce phase separation. The mixture was subjected to vigorous shaking for 1 min and then centrifuged at 3000 rpm for 5 min. For the clean-up step, 5 mL of acetonitrile solvent was added to a tube with sulfate magnesium and C18. The tube was then subjected to vigorous shaking for 1 min, after which it was centrifuged at 3000 rpm for 5 min at 4 °C. One milliliter of the solvent upper layer was transferred to a vial for subsequent chromatographic analysis.

Chromatographic analysis was conducted using an Agilent 1200 HPLC instrument (Agilent Technologies, Santa Clara, USA) coupled with a 4620 series ESI triple quadrupole MS/MS. A previously developed chiral method for rice grain (Cervantes-Díaz et al. 2024) was modified to enable the determination of profoxydim isomers in soil and their subsequent individual isolation. The separation was conducted using a Chiralcel OJ-3R column at 25 °C. The herbicide was eluted using an isocratic method of water acidification with 0.1% formic acid (A) and acetonitrile (B) with 60% B. The mobile phase flow rate was 1.0 mL/min and the injection volume was 5 µL.

The electrospray conditions were the following: the temperature of the drying gas was 300 °C; the flow rate of the drying gas mobile phase was 11 L/min; the pressure of the nebulizer was 40 psi; and the voltage of the capillary was 4000 V. The nitrogen generator Zefiro 35 was used as the nebulizer gas.

In order to achieve the greatest sensitivity for the precursor ion, the collision-induced fragmentation technique was optimized at the source by means of the fragmentor voltage. Subsequent MS/MS spectra were acquired in the product ion mode in order to gain insight into the fragment ions present. Upon determining the specific product ions for the herbicide, a Multiple Reaction Monitoring experiment was conducted in order to identify the optimal collision energy for each specific transition. The data were processed using Agilent Mass Hunter software. Subsequently, the concentration of profoxydim was calculated from the appropriate standard calibration curve, using the peak areas measured for the samples.

### Method validation

The method was validated in accordance with the conventional validation procedure outlined in the guidelines SANTE 11312/2021 (European Commission 2021a) and SANTE/2020/12830 (European Commission 2021b). This included the following parameters: specificity, linear range, limit of detection (LOD) and limit of quantification (LOQ), matrix effect, accuracy, and precision.

The linearity of the method was determined by analyzing standard solutions and different matrix-matched standard solutions in duplicate at eight concentrations, ranging from 0.00625 to 0.625 µg/mL for each stereoisomer. The parameters of the linear regression equations, including the slope, intercept, standard deviations, and the correlation coefficient (*R*^2^), were calculated based on an acceptance criterion of *R*^2^ higher than 0.99 and linearity residuals less than 30%.

The recovery assays were conducted to investigate the accuracy and precision of the method. Five replicates of the spiked samples at different levels (25.0, 50.0, 500.0, and 1000.0 µg/kg) were prepared on three different days. The target compounds were extracted and purified according to the aforementioned procedure. The precision in these conditions for repeatability, expressed as the relative standard deviation (RSD), was determined by the intra- and inter-day assays.

To ascertain the accuracy of the method, the recovery factor was required to lie within the range of 70 to 120%. With regard to precision, the relative standard deviation was to be less than 20%. Selectivity was established by extracting and analyzing a blank soil sample under identical conditions to identify any interfering peaks. The selected ion chromatography characteristic of each analyte was monitored during these analyses. The matrix-dependent limit of detection (LOD) and limit of quantification (LOQ) of the method were determined using the blank and calibration standards of the soil matrix. The LODs of the four stereoisomers were defined as the concentration that produced a signal-to-noise (peak-to-peak) ratio of 3, whereas the LOQs were defined as the concentration that produced a signal-to-noise ratio of 10; the LOQs are estimated from the chromatogram corresponding to the lowest point used in the matrix-matched calibration.

The matrix-induced signal suppression/enhancement (SSE) was determined by the slope ratio of the matrix-matched calibration curve/pure solvent calibration curve according to Eq. 1.

1 $$\text{Matrix effect }\left(\%\right)=\left(\frac{{S}_{m}}{{S}_{S}}-1\right)\times 100$$

where *S*_m_ is the soil matrix slope and *S*_S_ is the extraction solvent slope. In general, a matrix effect of ± 20% is regarded as exerting a considerable influence on the effectiveness of the analytical method (European Commission 2021b).

### Isolation of pure profoxydim isomers and soil dissipation studies

An Agilent HPLC–DAD model 1200 system model (Agilent Technologies, USA) equipped with a thermostatic column module, a quaternary pump, a diode array detector, and a fraction collector was used to isolate pure single profoxydim isomers. The previously described chromatographic method was used at a wavelength of 230 nm. Injections of 600 µL of a water/methanol (80/20) solution containing 500 mg/L of profoxydim standard were performed. To obtain sufficient amounts of profoxydim isomers,100 injections with 25-min cycle time were conducted. During the injections, the profoxydim isomers were collected separately in 20-mL tubes in the fraction collector. The collected fractions were pooled and the acetonitrile was evaporated with a stream of nitrogen. The aqueous phase was passed through a copolymer solid phase extraction cartridge, Isolute ENV + (1 g/6 mL) (Biotage, UK), to remove the formic acid and to extract the analytes. The elution of the compounds was achieved with 3 × 3 mL CH_2_Cl_2_. The organic extracts were dried over Na_2_SO_4_ and evaporated to dryness and the isomeric purity was determined by HPLC–DAD.

Dissipation studies were carried out in a climatic chamber in the dark at a temperature of 25 ± 2 °C and a relative humidity of 80 ± 1% (Binder KBWF 240, Germany), using 4 g of soil per 50-mL centrifuge tube, with ultrapure water added to simulate the maximum water holding capacity of the soil in flooded field conditions. Each isomer was applied at a concentration of 1.0 µg/g. In order to maintain a constant soil moisture content throughout the duration of the study, ultrapure water was added to each tube at regular intervals. A total of 150 tubes (3 replicates × 10 time intervals × 5 compounds) were used in this experiment.

To detect and follow the evolution of DPs, at different time intervals, samples were removed and analyzed by HPLC-QTOF-MS/MS. The optional separation conditions were established by injecting 5 μL, and a gradient program with a 0.7 mL/min flow rate was devised for detecting and monitoring their evolution. The mobile phase consisted of 0.5% formic acid (mobile phase A) and ACN (mobile phase B). The gradient started at 23% B, linearly increased to 62% B during 3 min, maintained for 14 min, increased to 90% B during 2 min, and then maintained for 5 min. The mobile phase was then returned to 23% B for reequilibration. The mass spectrometer was tested in positive and negative ion modes and was set up with the following optimized conditions for the target analytes: ion spray voltage of 5500 V, ion source gas 1 and gas 2 setting of 50 and 55 psi respectively. The curtain gas was set to 20 psi, with a declustering potential of 70 V and a declustering potential of 2 of 15 V. The analysis data obtained were processed using Analyst software (Applied Biosystems).

### Data analysis

The kinetic behavior of profoxydim was fitted to a first-order model (SFO), which postulates that the rate of decline in herbicide concentration is directly proportional to the remaining herbicide concentration in the system at any given time. This relationship is expressed by the following equation:

2 $$C(t)={C}_{0}\times \text{exp}(-kt)$$

The kinetics of DPs is more complex than that of the parent, due to the simultaneous occurrence of formation and degradation processes. The kinetic behavior was evaluated using the Computer Assisted Kinetic Evaluation (CAKE) Software, version 3.6, which enabled the data to be fitted to a different kinetic model. The software permits the fitting of a model not only for the dissipation of the active substance but also for the formation of DPs, where a single first-order model is considered for all individual pathways in the CAKE tool. In accordance with the recommendations of the FOCUS Degradation Kinetics Working Group (FOCUS 2006), the link between formation and degradation processes is established by utilizing metabolite formation fraction parameters to divide the overall degradation rate of the precursor between the metabolites formed. This is expressed by the following equation that describes the overall process for the evolution of each metabolite:

3 $$\frac{d(\text{Metabolite})}{dt}= {k}_{\text{parent}}\times {ff}_{\text{metabolite}}\times \text{Parent}- {k}_{\text{metabolite}}\times \text{Metabolite}$$

where *k*_parent_ is the rate constant of the parent, *ff*_metabolite_ is the formation fraction of the metabolite, and *k*_metabolite_ is the rate constant of the metabolite. Furthermore, the sum of the formation fractions of the metabolites formed from a single parent molecule should not exceed 1.

The diastereoselectivity of a degradation process can be quantified from the diastereomeric ratio (DR), using the following formula for each diastereoisomer of the profoxydim:

4 $$DR(\text{Isomer }X)= \frac{\text{Isomer }X}{\text{Isomer }1+\text{Isomer }2+\text{Isomer }3+\text{Isomer }4}$$

A variety of ANOVA tests were conducted in order to assess the influence of differing conditions on the determination of profoxydim. The statistical analyses were performed using the Statgraphics v.19 software (Statgraphics Technologies, Inc., USA).

## Results and discussion

### Efficiency of different sample pretreatment techniques

**Fig. 2:**
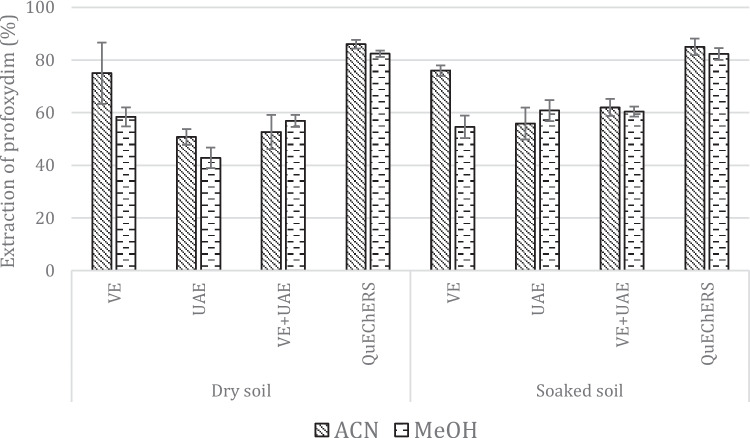
Recoveries of profoxydim for the VE, UAE, VE + UAE, and QuEChERS methods in dry and soaked soil samples

The pretreatment step of a method that includes extraction and clean-up steps is critical for analyzing pesticides in soil. Since no differences in the extraction step occurred for chiral compounds, we employed the analytical standard of profoxydim. Four extraction techniques were evaluated for their ability to extract profoxydim from soil: ultrasound-assisted extraction (UAE), vortex extraction (VE), a combination of VE and UAE (UAE + VE), and the QuEChERS method. These techniques were selected based on their simplicity, rapidity, and low solvent consumption, as well as their previous success in extracting pesticides from soils (Tadeo et al. 2010; Guerra et al. 2018; Castineira-Landeira et al. 2023; Liu et al. 2024). The extraction solvent is a key factor affecting the extraction recovery of solid samples. Thus, we chose two common liquid chromatography-compatible solvents: acetonitrile (ACN) and methanol (MeOH). We employed two soils: soaked soil to simulate common flooding irrigation practices and dry soil to mimic nonflooding management practices that have been used recently in the last decade to reduce water consumption (Surendran et al. 2021). Four grams of soil was placed in a 50-mL PTFE centrifuge tube and fortified with 1.0 µg/g of profoxydim. The extraction volume was 10 mL and three replicates were performed for each technique. The UAE method consists of applying ultrasound energy to extract liquid or solid samples. The samples were placed in an ultrasonic bath for 10 min. The second method corresponds to VE, and the samples were agitated for 10 min to rotate the fluids and create a vortex to extract the analyte from the soil. Then, the mixture was subjected to VE followed by UAE under the same conditions. All these methods were kept constant at room temperature. Finally, the samples subjected to these methods were centrifuged at 3000 rpm for 5 min at 4 °C. Figure 2 shows a comparison of the efficiencies of the four extraction methods employing acetonitrile or methanol.

The best results for both dry and soaked soil were obtained, in general, with acetonitrile. Recoveries with soaked soils seem to be greater when VE, UAE, and VE + UAE are used, probably due to the solubility of profoxydim in water, which favors extraction. Although VE achieved better recoveries using acetonitrile (75 ± 8%) than UAE and VE + UAE, the QuEChERS method demonstrated higher sensitivity and better extraction results with recovery values of 86 ± 1% and 84 ± 3% for dry and soaked soil, respectively. Moreover, the QuEChERS method offers additional advantages, including enhanced repeatability (lower RSE) due to its automated nature and the ability to process a greater number of samples simultaneously compared to VE. It is also important to acknowledge that the QuEChERS technique incurs an additional expense due to the salts that are not present in other methods.

### Optimization of the QuEChERS method

Once the QuEChERS technique was selected, several parameters that affect the extraction performance and efficiency were investigated. Thus, we evaluated the following parameters: (a) the extraction QuEChERS, non-buffered and buffered citrate and acetate; (b) the amount of sample, 2 and 5 g of soil; (c) the extraction solvents, methanol and acetonitrile; and (d) four purification sorbents, PSA, PSA-GCB, PSA-C18, and C18. All experiments were performed in triplicate with a spiking level of 1.0 µg/g.

Table 1 illustrates the recoveries observed for each of the investigated processes. Given that chiral compounds exhibit identical behavior in achiral media (e.g., as sorbents and solvents), the recoveries for the four stereoisomers can be considered to be identical. Consequently, Table 1 presents the recoveries of profoxydim as the sum of those of the four stereoisomers.

**Table 1:** Mean recoveries (RSD %) of profoxydim in paddy soil using different QuEChERS in the extraction step

Mean recoveries (RSD %); *n* = 3					
Type of soil		Soaked		Dry	
Amount of soil (g)		2	5	2	5
ACN	Citrate	81.68 (4.74)	72.08 (1.36)	85.14 (6.28)	70.64 (3.66)
	Acetate	31.82 (1.94)	83.25 (4.36)	51.25 (4.74)	65.18 (7.22)
	Non-buffered	88.42 (3.19)	77.27 (5.91)	102.82 (1.62)	80.86 (7.74)
MeOH	Citrate	57.58 (2.80)	44.17 (1.85)	46.52 (4.69)	59.86 (4.68)
	Acetate	9.18 (2.54)	12.25 (4.32)	13.13 (5.64)	18.14 (3.75)
	Non-buffered	60.91 (2.72)	66.98 (3.80)	49.50 (3.06)	58.14 (1.75)

An ANOVA test was conducted to ascertain whether statistically significant differences existed between the various conditions. The results indicated that there were significant differences in the recoveries when employing ACN versus MeOH (*p*-value = 0.0007). It was observed that ACN yielded superior results, with recoveries exceeding 70% in all the experiments. The use of either 2 g or 5 g of soil did not result in any significant differences (*p*-value = 0.8326), indicating that this particular parameter was not a limiting factor. When the QuEChERS sorbents, with ACN as the solvent, were compared, the non-buffered version presented the best performance in all cases, with recoveries above 77%. In conclusion, the best extraction conditions were obtained with ACN and the non-buffered version. We chose 2 g of soil because of its lower sample quantity.

Traditionally, an adequate dispersive-SPE clean-up step is essential for extracting pesticides from soil prior to HPLC trace analysis to achieve lower detection limits and high sensitivity (Lesueur et al. 2008; Rashid et al. 2010; Caldas et al. 2011). After extraction, the extract solution of soil typically contains humic acid and other interferents so a further clean-up procedure should be performed to reduce the matrix effect.

In the literature, the most typical clean-up sorbents used in soil samples to detect pesticides are PSA (Liu et al. 2024), C18, and GCB (Caldas et al. 2011; Abdel-Ghany et al. 2016). As shown in Table 2, the recoveries of the profoxydim moieties were very poor (below 30%) when PSA was used as the sorbent. The findings are consistent with those of other researchers who have observed that PSA binds strongly to a range of compounds including carboxylic acid groups (Abdel-Ghany et al. 2016; Lee et al. 2016). Conversely, the recoveries and RSDs were good when the C18 sorbent was employed in both mentioned matrices. Therefore, the non-buffered version and C18 for clean-up were chosen for the determination of profoxydim in soil.

**Table 2:** Mean recoveries ± RSD (%) of profoxydim in 2 g of paddy soil after extraction with ACN-non-buffered QuEChERS with four different clean-up methods

Type of soil	Mean recoveries (RSD %); *n* = 3			
	PSA	PSA-C18	PSA-GCB	C18
Soaked	10.49 (2.90)	15.09 (2.57)	27.40 (1.23)	98.40 (0.95)
Dry	16.69 (1.95)	28.46 (0.35)	8.46 (5.27)	102.92 (1.03)

### Method validation

The intensity signal attained in the positive mode was much higher for profoxydim isomers than in the negative mode. The most intense and consistent product ion MS spectra were obtained by optimizing the declustering potential and collision energy.

The four compounds exhibited a quantified transition at m/z 466.1 > 280.1 selected to quantify, and that corresponds to the loss of the oxime, –C_9_H_10_O_2_Cl, with a declustering potential of 120 V, a collision energy of 12 eV, and a transition at m/z 466.1 > 180.1 selected to confirmation purposes with a declustering potential of 120 V and a collision energy of 25 eV.

#### Matrix effect, linearity, LOD, and LOQ

A major drawback in the analysis of pesticide residues in complex samples by LC–MS is represented by the occurrence of matrix effects, which is considered to be an unexpected suppression or enhancement of the analyte response due to coeluting sample constituents that will significantly influence the quantity of ionized analyte molecules that reach the MS/MS path (Paya et al. 2007). Generally, the suppression or enhancement effect is originated from the insufficiently removing endogenous compounds though the real mechanism underlying these matrix effects is still not fully understood. Therefore, in the current study, the matrix effect on the MS/MS detector using the proposed method was investigated in soaked and paddy soils by comparing the standards in the solvent with the matrix-matched standards. Therefore, no external matrix-matched standards were needed and a calibration curve was performed in acetonitrile.

The linearity, LOD, and LOQ were obtained using the peak areas of the product ions obtained from the MS/MS mode. The linearity was evaluated by preparing different calibration curves in acetonitrile within the concentration range of 0.00625 to 0.625 µg/mL for each isomer. Satisfactory linearities were observed for all stereoisomers (*R*^2^ ≥ 0.997 in all cases).

The developed LC–MS/MS method was evaluated in accordance with the above-mentioned European Union guideline (European Commission 2021b). The LOQs were estimated as the minimum concentration of a compound giving a peak with a signal/noise ratio of not less than 3:1. The estimated LODs for the four isomers were determined to be 7.6 µg/kg, while the LOQs were established to be 25.0 µg/kg.

#### Precision and accuracy

The recovery and RSDs of the isomers were measured to validate the chiral LC–MS/MS method by spiking the blank samples with three different concentrations (25.0, 50.0, 500.0, and 1000.0 µg/kg) and then analyzing them in quintuplicate (Table 3). Additionally, two unfortified control samples were subjected to analysis.

**Table 3:** Recoveries (*n* = 5, %) and RSD (%) of the target profoxydim isomers in dry and soaked soil matrices at three spiked levels. ª*n* = 6; ^b^*n* = 3

Matrix	Isomer	Fortification level (µg/kg)	Mean recovery (%)	Intra-day (RSD)^a^ (%)	Inter-day (RSD)^b^ (%)
Dry paddy soil	Isomer 1	25.0	88.27	4.53	9.67
		50.0	95.96	4.95	10.50
		500.0	104.93	3.42	10.59
		1000.0	105.70	4.32	11.02
	Isomer 2	25.0	91.17	2.60	6.42
		50.0	96.31	2.07	8.55
		500.0	104.33	4.41	11.14
		1000.0	103.61	3.85	10.45
	Isomer 3	25.0	91.14	1.59	10.09
		50.0	93.16	2.43	4.59
		500.0	104.43	3.06	10.79
		1000.0	105.45	3.87	9.65
	Isomer 4	25.0	91.29	5.33	9.34
		50.0	91.34	3.25	8.58
		500.0	104.71	2.94	9.65
		1000.0	104.95	4.23	10.50
Soaked paddy soil	Isomer 1	25.0	99.80	6.74	7.40
		50.0	106.40	7.73	4.90
		500.0	101.90	5.35	8.14
		1000.0	103.86	6.04	11.20
	Isomer 2	25.0	99.91	4.36	8.10
		50.0	101.33	5.44	6.14
		500.0	104.59	6.03	4.32
		1000.0	105.22	5.68	9.02
	Isomer 3	25.0	100.20	4.40	7.30
		50.0	106.53	5.51	6.82
		500.0	98.25	6.40	2.21
		1000.0	101.56	4.95	8.14
	Isomer 4	25.0	105.49	2.28	3.15
		50.0	105.92	3.59	6.39
		500.0	102.75	6.16	3.94
		1000.0	103.23	5.42	7.25

The precision of the method was determined by the repeatability and reproducibility studies and expressed as the RSD. The intra-day precision was measured by comparing the standard deviation of the recovery percentages of the spiked samples at three fortification levels run during the same day. The inter-day precision was determined by analyzing the spiked samples for three consecutive days. As Table 3 shows, the method presented satisfactory mean recovery values ranging from 88.27 to 104.93% for dry soil and from 98.25 to 106.53% for soaked soil. For precision, all intra-day RSD for the isomers was found to be within the range of 1.59–5.33% for dry soil and 2.28–7.73% for soaked soil. Regarding inter-day RSDs, the values ranged from 2.21 to 11.14% for the four isomers in both matrices.

These outcomes are in accordance with the recommendations set out by the European Commission (European Commission 2021a and European Commission 2021b) for recovery in chiral LC–MS/MS stereoselective methods, which specify an average recovery range of between 70 and 120% and an RSD of no more than 20%. The results demonstrate that this method is an effective tool for the stereomeric analysis of profoxydim in soil, as it achieves satisfactory precision and accuracy.

### Dissipation studies

#### Isolation of pure profoxydim isomers

To investigate the degradation of each single isomer of profoxydim, it was necessary to obtain adequate quantities of each compound due to the lack of analytical standards. Consequently, the isomers were isolated chromatographically. Since there are no semipreparative or preparative columns commercially available for the Chiralcel OJ-3R column, consecutive runs were performed by injecting 600 µL of 500 mg/L and collecting each of the four compounds. Two types of solid phase extraction (SPE) sorbents were tested for their ability to extract and concentrate the compounds from the collected aqueous phase: C18 cartridges and a hydroxylated highly cross-linked styrene/divinylbenzene copolymer, Isolute ENV + . Different eluents, including ACN, MEOH, and CH_2_Cl_2_, were evaluated with different elution volumes to elute the compounds from the sorbent. The results showed that C18 did not retain profoxydim, and the recoveries were less than 60%. The optimal extraction conditions were found to be ENV + and elution with 3 × 3 mL CH_2_Cl_2_. We finally obtained 10 mg of each isomer.

#### Kinetic degradation of pure isomers of profoxydim in soil

**Fig. 3:**
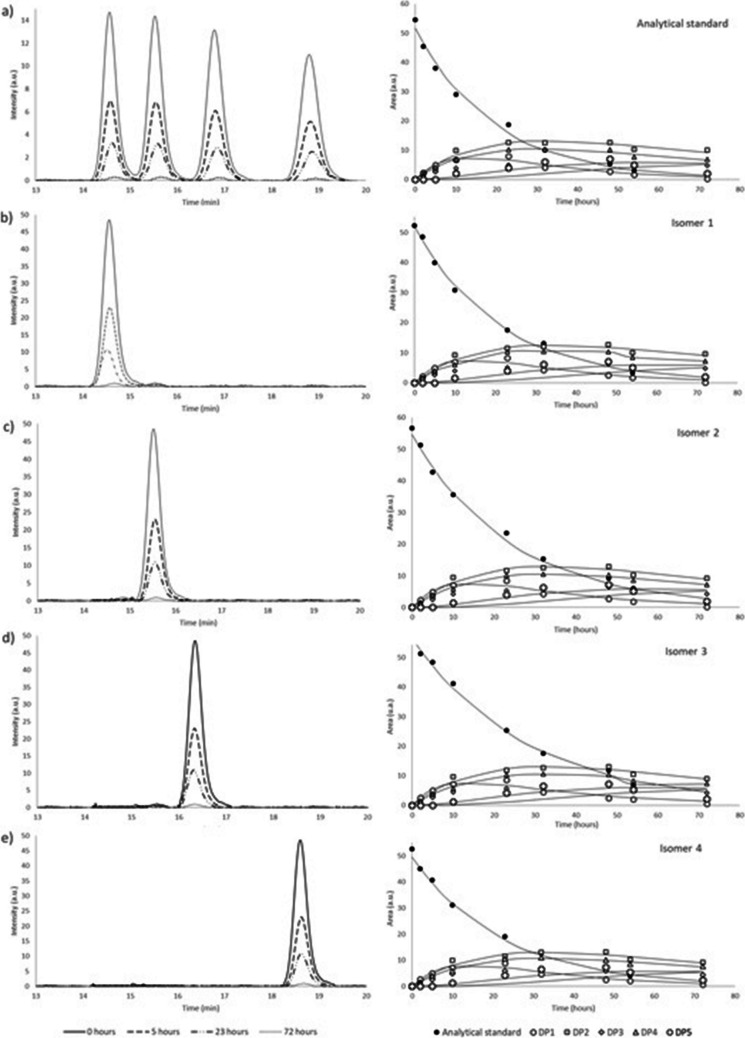
Kinetic evolution and chromatograms of profoxydim and isolated isomers and their evolution of DPs. **a**) Analytical standard of profoxydim; **b**) isomer 1; **c**) isomer 2; **d**) isomer 3; **e**) isomer 4

In a previous study, we investigated the biotic degradation of profoxydim as a mixture of the four isomers (Cervantes-Diaz et al. 2024). Our findings indicated that the biodegradation of all profoxydim isomers occurred rapidly, with half-lives ranging from 0.47 to 0.53 days. However, ignoring stereoselectivity may result in an over- or under-estimation of their environmental fate. Consequently, the individual data of each isomer is of the utmost importance for the purpose of obtaining more precise data for the performance of accurate risk assessment. It has been demonstrated that individual stereoisomers degrade at different rates (Buerge et al. 2003) playing the microbial communities a key role in the enantioselective degradation of pesticides (Wang et al. 2005; Wong 2006). Therefore, separate incubations of samples with individual isomers were performed to determine their dissipation behavior. In addition, interconversion between enantiomers may occur, as has been observed with other chiral compounds such as the herbicide haloxyfop (Poiger et al. 2015), so our aim is also to detect any potential enantiomerization and/or enantioconversion of the compound. Figure 3 shows the chromatograms and kinetic degradation for the standard of profoxydim and for each isomer until the complete dissipation of profoxydim.

The degradation of the profoxydim isomers followed an exponential decline and was adequately described with the SFO model with *R*^2^ values above 0.992, which indicates a good fit.

The four isomers were degraded considerably quickly, with half-lives ranging from 14.7 (isomer 1) to 19.5 (isomer 3) h. After 70 h, the isomeric forms were completely degraded. The selective degradation of profoxydim in paddy soil was low. The preferential degradation followed the order of isomer 1 > isomer 4 > isomer 2 > isomer 3. ANOVA was performed to compare the degradation of the four isomers, and we found significant differences between isomers 1 and 4 and between isomers 2 and 3 (Table 4). Nevertheless, the disparity in the half-lives of the isomers is minimal, likely due to the predominant role of chemical processes over biological degradation, which are non-enantioselective throughout the degradation process.

**Table 4:** First-order kinetic parameters for the degradation of the profoxydim and isolated isomers

Parameter	Rac-profoxydim	Isomer 1	Isomer 2	Isomer 3	Isomer 4
*C*_0_ (mg/L)	51.64 ± 1.37	52.01 ± 0.84	54.62 ± 1.60	56.46 ± 1.01	49.46 ± 1.27
*k* (h^−1^)	0.042 ± 0.003	0.047 ± 0.002	0.041 ± 0.003	0.035 ± 0.001	0.044 ± 0.003
*R*^2^	0.992	0.997	0.994	0.995	0.992
Half-live (hours)	16.5 ± 0.4	14.7 ± 0.6 ^a^	16.8 ± 0.6 ^b^	19.5 ± 0.5 ^c^	15.6 ± 0.6 ^a^
DR at 23 h	-	20.5	27.5	29.7	22.3
DR at 54 h	-	16.3	30.1	33.5	20.2

The diastereomeric ratio (DR) was determined to measure the diastereoselectivity of profoxydim in soil degradation. The DR represents the proportion of each isomer’s composition, calculated using Eq. 3 (Krupčík et al. 2015; Herrera et al. 2019). After 23 h of degradation (Table 4), isomers 1 and 4 exhibited lower DRs (20.5% and 22.3%, respectively), indicating faster degradation. Consequently, a diastereomeric excess of isomers 2 and 3 was observed at that time. Additionally, the DR was calculated at 54 h (Table 4), and isomers 1 and 4 exhibited similar behavior, with DR values of 16.3% and 20.2%, respectively, due to their rapid degradation.

As the degradation time increased, the DRs of isomers 2 and 3 also increased, as they degraded at a slower rate. Therefore, profoxydim shows stereoselectivity, with a preference for degrading isomers 1 and 4. No interconversion of enantiomers was observed, as a single peak/isomer was detected in each of the separate experiments. Therefore, any change in the stereomeric composition of the profoxydim enantiomers is due to differences in the degradation rates of the individual isomers and not to the interconversion of enantiomers.

Further studies should be carried out in other soil types with different physicochemical and microbial properties to understand the relationships between soil characteristics and enantioselective degradation. This will improve our understanding of the fate of profoxydim isomers in the environment.

#### Study and evolution of DPs

**Fig. 4:**
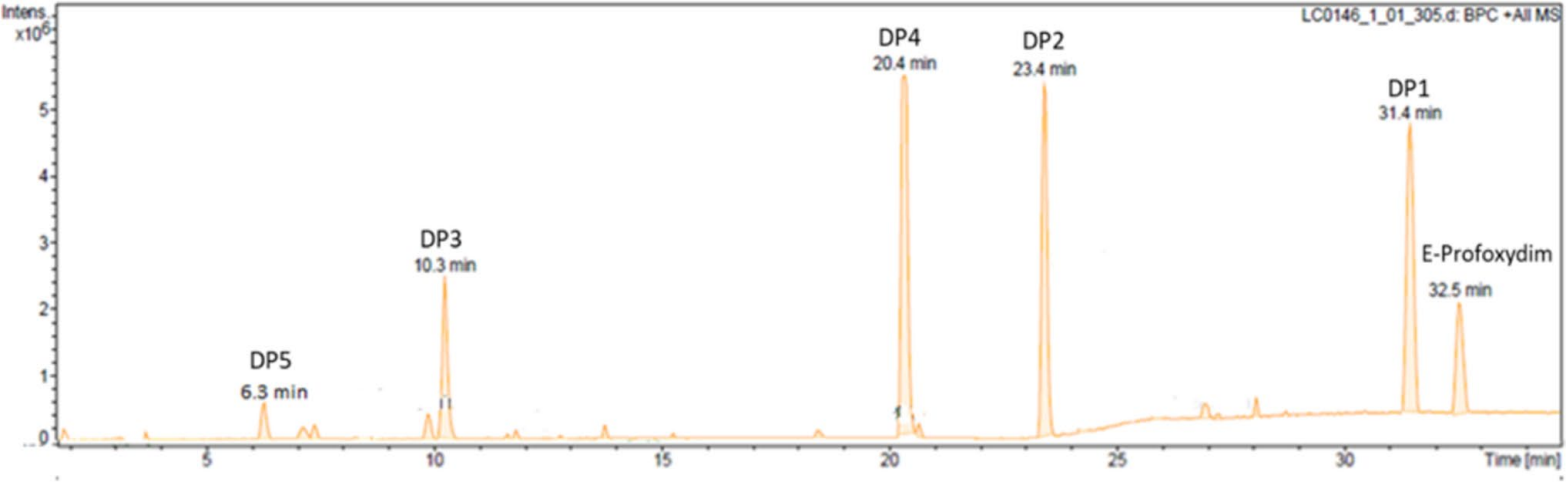
Total ion chromatogram of the degradation of one of the profoxydim isomers

DPs for each isomer were analyzed by HPLC-QTOF-MS/MS. Figure 4 shows a typical HPLC–MS chromatogram of the degradation of profoxydim isomers. Up to five DPs were detected in the four stereoisomers.

**Fig. 5:**
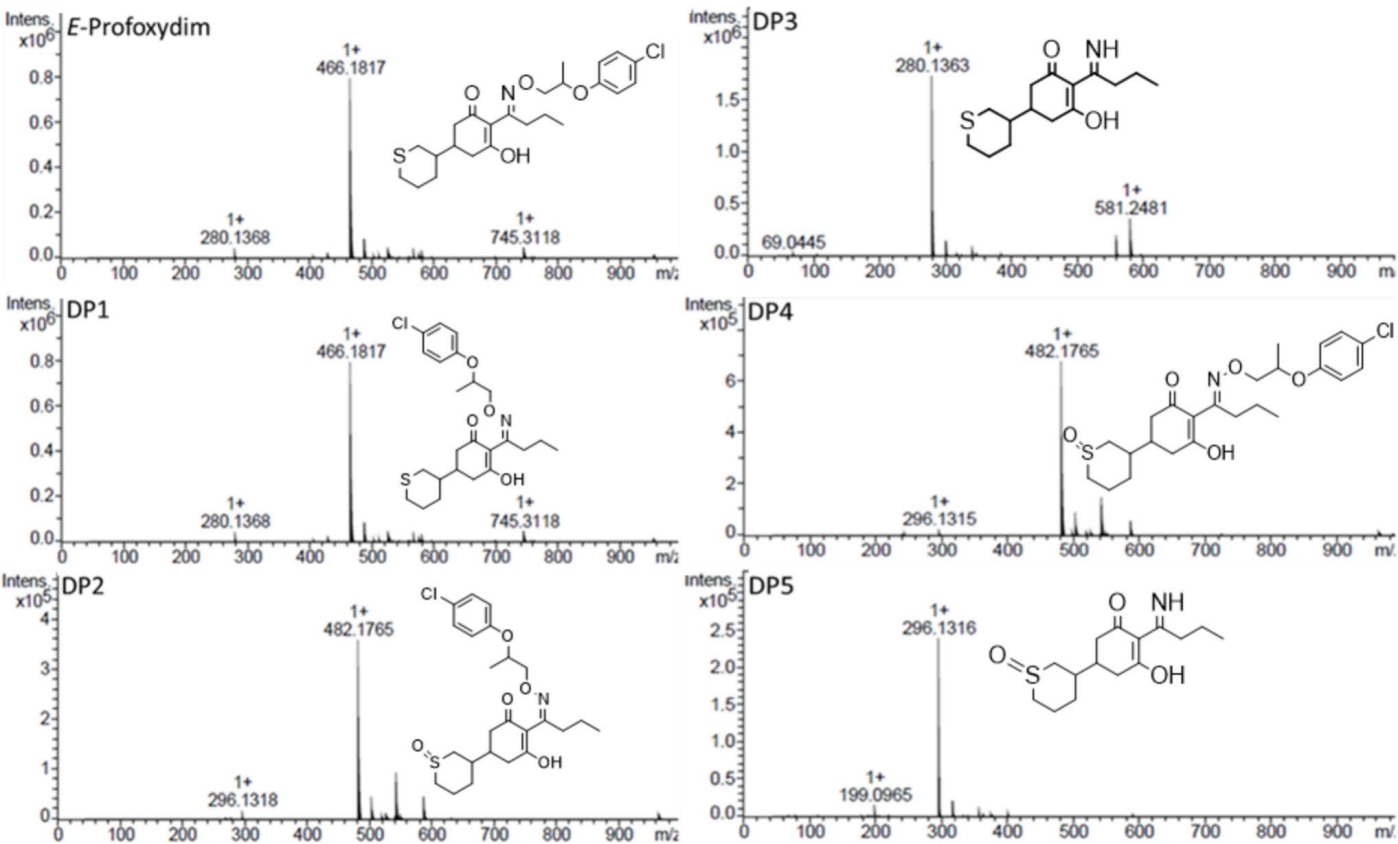
MS/MS spectra and chemical structures of profoxydim and its DPs

Figure 5 depicts the MS/MS of the DPs. DP1 has an *m/z* of 466.1817 and a retention time of *R*_t_ = 31.4 min and corresponds to the *Z*-isomer of the profoxydim from the E parent compound, with half-lives ranging from 5.44 to 6.99 h (Table 5).

**Table 5:** Kinetic parameters of profoxydim degradation products

DPs	Parameters	Isomer 1	Isomer 2	Isomer 3	Isomer 4
	*R*^2^	0.978 ± 0.013	0.976 ± 0.025	0.971 ± 0.019	0.968 ± 0.022
DP1	*k* (h^−1^)	0.099 ± 0.045	0.112 ± 0.016	0.127 ± 0.009	0.101 ± 0.015
	*t*_1/2_ (h)	6.99 ± 0.23^c^	6.18 ± 0.41^b^	5.44 ± 0.52^a^	6.89 ± 0.33^c^
DP2	*k* (h^−1^)	0.017 ± 0.013	0.021 ± 0.010	0.025 ± 0.011	0.019 ± 0.009
	*t*_1/2_ (h)	41.6 ± 1.03^c^	32.1 ± 0.48^a^	38.8 ± 0.71^b^	33.2 ± 0.63^a^
DP3	*k* (h^−1^)	0.039 ± 0.024	0.046 ± 0.014	0.046 ± 0.011	0.041 ± 0.017
	*t*_1/2_ (h)	17.9 ± 0.12^c^	14.9 ± 0.22^a^	15.0 ± 0.09^a^	16.9 ± 0.23^b^
DP4	*k* (h^−1^)	0.018 ± 0.013	0.021 ± 0.007	0.024 ± 0.010	0.019 ± 0.009
	*t*_1/2_ (h)	37.5 ± 0.56^c^	31.1 ± 0.34^a^	38.1 ± 1.01^d^	35.7 ± 0.52^b^
DP5	*k* (h^−1^)	-	-	-	-
	*t*_1/2_ (h)	-	-	-	-

DP2 and DP4, with *R*_t_ values of 20.4 and 23.4 min, respectively, presented a *m/z* of 482.1765, which corresponds to 16 Da greater than that of profoxydim and was identified as the sulfoxide of profoxydim, the main DP. The oxidation of the sulfur atom of profoxydim implies the generation of a new chiral carbon, which results in the formation of eight stereoisomers (number of stereoisomers = 2^n^; *n*, chiral centers). The separation of these isomers could not be achieved with the chiral column employed for profoxydim, so an achiral C18 column was used to obtain good peak resolution. They presented half-lives between 32.1 and 41.6 and between 31.1 and 37.5 h, respectively (Table 5). This DP has been reported to be more persistent in soil than its parent (Sanchez et al. 2006; Cervantes-Diaz et al. 2024). DP3, with an *R*_t_ of 10.3 min and an *m/z* of 280.1363, corresponds to the imine-profoxydim group as a consequence of the cleavage of the oxime moiety. This compound has a half-life between 14.9 and 17.9 h. For other cyclohexanedione herbicides, such as clethodim (Sandín-España et al. 2016), alloxydim (Sevilla-Morán et al. 2008), or sethoxydim (Sevilla-Morán et al. 2017), the most common degradation products of various processes, such as photolysis and hydrolysis, are the sulfoxide when a sulfur atom is present in the structure and imine compounds. Finally, DP5, with an *R*_t_ of 6.3 min and an *m/z* of 296.1316, is a minor product and was identified as the imine of the profoxydim-sulfoxide, which is likely generated by the S-oxidation of the imine (in Fig. 1 of S1 a tentative degradation pathway is depicted).

The degradation of profoxydim isomers and their resulting products (DPs) was monitored until they fully disappeared. As shown in Fig. 3, the four stereoisomers followed nearly identical degradation patterns, similar to those observed in the herbicide standard reported by Cervantes et al. (2023). No quantitative statement can be made as no reference compounds are available, but only a semiquantitative method can be used. The data of DPs were fitted by the software CAKE (Table 5), which takes into account a global process involving the dissipation of the herbicide and the formation of the DP through the use of metabolite formation fraction parameters (FOCUS 2006). The detailed kinetic parameters are presented in Table 1 of the Supplementary Information. Due to the low concentration of DP5, it was not possible to obtain a good fit for this compound.

An ANOVA analysis was conducted to assess the significant differences in the degradation of each DP of isomers. The results indicate significant differences in the half-lives of the same degradation products among different isomers. Such differences may be attributed to the spatial configuration of the isomers, which could either promote or sterically hinder the chemical reactions involved in degradation. Although the data is statistically significant, it is not particularly relevant in terms of environmental impact (for example, 14.9 h vs 17.9 h). Overall, the *Z*-isomer emerges as the degradation product with the shortest half-life, displaying values below 7 h, followed by the imine byproduct, which has half-lives ranging from 14.9 to 17.9 h. The primary degradation product, sulfoxide, exhibits the longest half-lives, extending up to 41.6 h.

## Conclusions

In this study, on one hand, a chiral method for the simultaneous detection of the four isomers of profoxydim in paddy soil by means of QuEChERS and HPLC–MS/MS was validated. This method was developed according to the SANTE European guidelines, and the linearity, precision, accuracy (from 88.27 to 106.53% with RSD ≤ 14.11), LOD (7.6 µg/kg), and LOQ (25 µg/kg) were successfully determined, demonstrating the suitability of this stereoselective method.

On the other hand, we achieved, for the first time, the isolation of the four pure isomers of profoxydim to study their individual degradation. The distinct behaviors of the isomers were identified in paddy soil, which demonstrated rapid and stereoselective degradation with half-lives ranging from 14.7 to 19.5 h. Furthermore, there was no evidence of interconversion among the four stereoisomers. Moreover, the DPs generated followed the same pattern profile for the four isomers. The main degradation product profoxydim-sulfoxide was found to be more stable than the parent compound, indicating the importance of further studies in future works.

The data from this study can help to provide a sound scientific basis for improving the accurate risk assessment of profoxydim. Additionally, this work illustrates the need to characterize isomers of chiral agrochemicals in the environment to obtain an accurate understanding of their distribution and fate in the environment.

## Supplementary Information

Below is the link to the electronic supplementary material.

Supplementary file1 (DOCX 43 KB)

## Data Availability

All supporting data generated and/or analyzed during this study are available from the corresponding author upon request.
