# Supplementary material for: Chiral method validation and stereoselective degradation of profoxydim isomers in paddy soil
Source: Environ Sci Pollut Res Int. 2024 Nov 20;31(56):64987–5001. doi: 10.1007/s11356-024-35557-z (PMC11624230; doi:10.1007/s11356-024-35557-z)
Supplement: Supplementary file 1 — Supplementary file1 (DOCX 43 KB) [file 11356_2024_35557_MOESM1_ESM.docx]

Detailed information regarding the salts and quantities used in SPE and QuEChERS methods (Section 2.1. Chemicals and materials)

SPE cartridges from International Sorbent Technology (IST, Mid Glamorgan, UK).:

- Isolute ENV+: 500 mg/6 mL
- C18: 500 mg/6 mL

QuEChERS methods acquired from HPC Standards GmbH (Symta, Madrid, Spain)

- Citrate-buffered: 4.0 g magnesium sulphate, 1.0 g sodium chloride, 0.5 g sodium
- Citrate dibasic sesquihydrate, 1.0 g sodium citrate tribasic dihydrate
- Acetate-buffered: 6.0 g magnesium sulphate, 1.5 g sodium acetate
- Non-buffered method: 4.0 g magnesium sulphate, 1.0 g sodium chloride)

Adsorbents employed in the d-SPE clean-up step, from HPC Standards GmbH (Symta, Madrid, Spain)

- PSA-Mix 6, containing 150 mg of PSA,
- PSA-C18-Mix 6, containing 150 mg of PSA and 150 mg of C18
- PSA-GCB-Mix 14 with 100 mg of PSA and 30 mg of GCB
- C18-drug residue, containing magnesium sulphate and 150 mg of C18.

**Figure 1.** Proposed degradation route of profoxydim isomers in soil

**Table S1.** Kinetic parameters of global process involving the dissipation of profoxydim and the formation of the degradation products in Isomer 1.

| **Parameter** | **Value** | **σ** | **Prob. > t** | **Lower (90%) CI** | **Upper (90%) CI** | **Lower (95%) CI** | **Upper (95%) CI** |
| --- | --- | --- | --- | --- | --- | --- | --- |
| Profoxydim_0 | 52,01 | 0,9149 | n.r. | 50,47 | 53,55 | 50,15 | 53,87 |
| k_Profoxydim | 0,04727 | 0,002081 | 3,08E-023 | 0,04376 | 0,05078 | 0,04305 | 0,051 |
| f_Profoxydim_to_DP2 | 0,304 | 0,1114 | n.r. | 0,1159 | 0,4922 | 0,07798 | 0,53 |
| f_Profoxydim_to_DP4 | 0,2628 | 0,09149 | n.r. | 0,1083 | 0,4173 | 0,07726 | 0,448 |
| f_Profoxydim_to_DP1 | 0,4332 | 0,1653 | n.r. | 0,1541 | 0,7123 | 0,09795 | 0,768 |
| k_DP2 | 0,01667 | 0,01333 | 0,1095 | -0,005827 | 0,03917 | -0,01035 | 0,044 |
| f_DP2_to_DP3 | 1 | 278,8 | n.r. | -469,7 | 471,7 | -564,4 | 566,4 |
| k_DP3 | 0,03882 | 0,02433 | 0,4654 | -0,7097 | 0,7873 | -0,8603 | 0,938 |
| f_DP3_to_DP5 | 1 | 11,97 | n.r. | -19,2 | 21,2 | -23,27 | 25,27 |
| k_DP5 | 0,03503 | 0,1402 | 0,4021 | -0,2017 | 0,2718 | -0,2494 | 0,319 |
| k_DP4 | 0,01838 | 0,01306 | 0,08278 | -0,003563 | 0,04052 | -0,008 | 0,045 |
| f_DP4_to_DP3 | 1 | 288 | n.r. | -485,3 | 487,3 | -583,1 | 585,1 |
| k_DP1 | 0,09923 | 0,04544 | 0,01778 | 0,02252 | 0,1759 | 0,007082 | 0,191 |
